# Host-microbe computational proteomic landscape in oral cancer revealed key functional and metabolic pathways between *Fusobacterium nucleatum* and cancer progression

**DOI:** 10.1038/s41368-024-00326-8

**Published:** 2025-01-02

**Authors:** Camila Paz Muñoz-Grez, Mabel Angélica Vidal, Tamara Beatriz Rojas, Luciano Esteban Ferrada, Felipe Andrés Zuñiga, Agustin Andrés Vera, Sergio Andrés Sanhueza, Romina Andrea Quiroga, Camilo Daniel Cabrera, Barbara Evelyn Antilef, Ricardo Andrés Cartes, Milovan Paolo Acevedo, Marco Andrés Fraga, Pedro Felipe Alarcón-Zapata, Mauricio Alejandro Hernández, Alexis Marcelo Salas-Burgos, Francisco Tapia-Belmonte, Milly Loreto Yáñez, Erick Marcelo Riquelme, Wilfredo Alejandro González, Cesar Andrés Rivera, Angel Alejandro Oñate, Liliana Ivonne Lamperti, Estefanía Nova-Lamperti

**Affiliations:** 1 Molecular and Translational Immunology Laboratory, Department of Clinical Biochemistry and Immunology, Pharmacy Faculty, Universidad de Concepción, Concepción, Chile; 2 Facultad de Odontología y Ciencias de la Rehabilitación, Universidad San Sebastián, Concepción, Chile; 3 Department of Computer Science, Universidad de Concepción, Concepción, Chile; 4 Facultad de Ingeniería, Universidad de Talca, Talca, Chile; 5 CMA BIO BIO, Faculty of Biological Sciences, Universidad de Concepción, Concepción, Chile; 6 BIOTER Laboratory, Clinical Biochemistry and Immunology Department, Pharmacy Faculty, Universidad de Concepción, Concepción, Chile; 7 Department of Pharmacology, Faculty of Biological Sciences, Universidad de Concepcion, Concepción, Chile; 8 Facultad de Medicina y Ciencia, Universidad San Sebastián, Concepción, Chile; 9 MELISA Institute, San Pedro de la Paz, Chile; 10 Cancer Molecular Dynamics Laboratory, Pharmacology Department, Faculty of Biological Sciences, Universidad de Concepción, Concepción, Chile; 11 Anatomy Pathology Unit and Dental Service, Oral Pathology Department, Hospital Las Higueras, Talcahuano, Chile; 12 Respiratory diseases Department, Faculty of Medicine, Pontifical University Catholic of Chile, Santiago, Chile; 13 Dentistry Faculty, Universidad de los Andes, Santiago, Chile; 14 Center for Research and Innovation in Biomedicine, Universidad de Los Andes, Santiago, Chile; 15 Oral Medicine and Pathology Research Group, Faculty of Health Sciences, Universidad de Talca, Talca, Chile; 16 Laboratory of Molecular Immunology, Department of Microbiology, Faculty of Biological Sciences, Universidad de Concepción, Concepción, Chile

**Keywords:** Bacteria, Periodontitis, Oral cancer

## Abstract

Oral squamous cell carcinoma (OSCC) is the most common manifestation of oral cancer. It has been proposed that periodontal pathogens contribute to OSCC progression, mainly by their virulence factors. However, the main periodontal pathogen and its mechanism to modulate OSCC cells remains not fully understood. In this study we investigate the main host-pathogen pathways in OSCC by computational proteomics and the mechanism behind cancer progression by the oral microbiome. The main host-pathogen pathways were analyzed in the secretome of biopsies from patients with OSCC and healthy controls by mass spectrometry. Then, functional assays were performed to evaluate the host-pathogen pathways highlighted in oral cancer. Host proteins associated with LPS response, cell migration/adhesion, and metabolism of amino acids were significantly upregulated in the human cancer proteome, whereas the complement cascade was downregulated in malignant samples. Then, the microbiome analysis revealed large number and variety of peptides from *Fusobacterium nucleatum* (*F. nucleatum*) in OSCC samples, from which several enzymes from the L-glutamate degradation pathway were found, indicating that L-glutamate from cancer cells is used as an energy source, and catabolized into butyrate by the bacteria. In fact, we observed that *F. nucleatum* modulates the cystine/glutamate antiporter in an OSCC cell line by increasing SLC7A11 expression, promoting L-glutamate efflux and favoring bacterial infection. Finally, our results showed that *F. nucleatum* and its metabolic derivates promote tumor spheroids growth, spheroids-derived cell detachment, epithelial-mesenchymal transition and Galectin-9 upregulation. Altogether, *F. nucleatum* promotes pro-tumoral mechanism in oral cancer.

## Introduction

Head and neck squamous cell carcinoma (HNSCC) ranks among the top 10 most common cancers worldwide. Its incidence is around 4%-5% and its mortality describes 1.5% of all cancers^1^. Oral squamous cell carcinoma (OSCC), a subtype of HNSCC, is the most common oral manifestation of this cancer^2^. OSCC represents 90% of all oral malignancies cases, and it has a poor 5-year survival rate^3^. Despite advances in surgical management, chemotherapy and radiotherapy, these percentages have not improved^4^. Early diagnosis and management are crucial on the survival rate and the quality of life of patients with OSCC^5^. Its etiology is multifactorial, among its main etiological agents are long-term use of tobacco, alcohol abuse, sun exposure, premalignant oral lesions, viruses (HPV) and sexually transmitted diseases^6^. However, lately, it has been reported high incidence and aggressive behaviors of OSCC, not fully explained by these risk factors, thus oral dysbiosis related to poor oral hygiene or periodontitis has been proposed as an important agent participating in OSCC tumor development, progression and metastasis^7–9^. Periodontitis is a chronic inflammatory disease with multiple contributing factors, linked to the formation of dysbiotic plaque biofilms. It is marked by the gradual deterioration of the structures supporting the teeth^10^. In this condition, specific bacteria have been involved, such as the red-complex (*Porphyromonas gingivalis*, *Tannerella forsythia*, *Treponema denticola*) and the orange-complex (*Fusobacterium nucleatum*, *Prevotella intermedia, Prevotella nigrescens, Peptostreptococcus micros, Streptococcus constellatus, Eubacterium nodatum, Campylobacter showae, Campylobacter gracilis, and Campylobacter rectus)* bacteria^11,12^. Microorganisms from the mouth are among the most abundant in the human body, and they are essential for sustaining a healthy oral environment^13^. Consequently, imbalance in the microbiome of susceptible individuals can result in both oral and systemic diseases, including periodontitis or cancer^13,14^.

The association of oral cancer and periodontitis has been explained by the large number of periodontal bacteria detected on the OSCC tissues^15^. Through the advent of high-throughput, next-generation sequencing (NGS), several studies have assessed to bacterial profiles associated with OSCC, detecting a group of periodontitis-related species enriched on tumoral tissues compared to controls samples^16–18^. It has been reported that some species could survive from both superficial and deep portions of OSCC^19^ and their products such as enzymes (phospholipase, proteases, fibrinolysin, collagenases), metabolic byproducts (fatty acids, ammonia, hydrogen sulfide) and endotoxins (lipopolysaccharides) could be toxic to oral epithelial cells, inducing mutations or altering signaling pathways that participate on proliferation and/or survival of cancer cells^20^, however specific tumorigenic bacteria and the precise mechanisms by which they influence oral cancer progression are still unclear.

According to previous metatranscriptomic analysis of the oral microbiota associated with OSCC sites in humans, only *Fusobacterium nucleatum* (*F. nucleatum*) was highly active in OSCC tissues^18^.

*F. nucleatum* is a gram-negative, anaerobic, opportunistic bacteria^21^ associated with diverse systemic diseases^22,23^, including cancer. Its role as an oncogenic bacterium has been mostly studied on colorectal cancer^24^ and the mechanisms are related to its main virulence factors^25^. However, researchers are presently investigating the precise mechanisms by which periodontal bacteria, such as *F. nucleatum* influence oral cancer development.

In vitro and in vivo studies have proposed that periodontal bacteria could alter tumor mass and promote tumoral growth by increasing the proliferation of OSCC cells^26,27^ either by altering cell cycling^7,28^ or causing DNA damage^27^. Also, they have been related to epithelial mesenchymal transition (EMT) by promoting the expression of EMT-classical markers^29–33^. It has been shown that periodontal bacteria modulate EMT phenotype in oral cancer cells by upregulating the expression of metalloproteinases (MMP-9, MMP-1, MMP-2, MMP-10)^29,34^, transcription factors (STAT-3, ZEB-1, MYC, Snail)^32^ and downregulating the expression of epithelial markers (E-cadherine, Cytokeratine 13)^33^. Even, the presence of these bacteria on OSCC could modulate local immunity, upregulating cytokines such as IL-8, IL-6, TNF-α, IL-1a, polymorphonuclear leukocytes (PMNs) and myeloid-derived suppressor cells (MDSCs) promoting an oral proinflammatory environment^35^ with a suppressed immune response which is permissive for tumor progression^36^. In OSCC the presence of immunosuppressive molecules or immune-checkpoint inhibitors is associated with early metastasis, degree of differentiation, lymph node invasion and poor diagnosis^37–42^.

In this study, we analyzed the main host-pathogen pathways in the secretome of biopsies from patients with OSCC and healthy controls by performing a computational proteomics landscape. Then, we evaluated the protumoral mechanisms and metabolic pathways of the main OSCC-associated bacteria.

## Results

### Host and microbe computational proteomic landscape in oral cancer

**Fig. 1:**
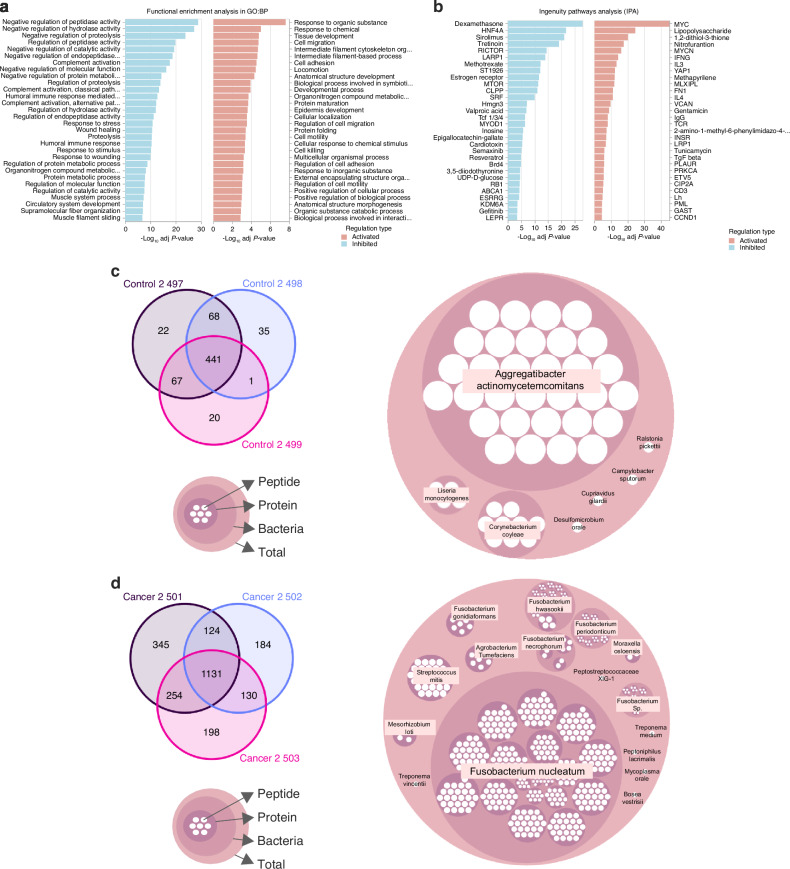
Computational proteomic analysis of host-microbe interactions in oral cancer. **a** Gene ontology functional enrichment analysis in biological process (GO:BP) and **b** Upstream regulator analysis in Ingenuity Pathways Analysis (IPA) of activated (red) and inhibited (blue) pathways identified in human malignant samples in comparison with healthy samples. **c** Venn diagram of peptides from healthy samples triplicates and the identification of unique healthy-samples proteins (pink circle) with the corresponding peptides (white circle) from specific bacteria. **d** Venn diagram of peptides from malignant samples triplicates and the identification of unique malignant-samples proteins (pink circle) with the corresponding peptides (white circle) from specific bacteria

**Fig. 2:**
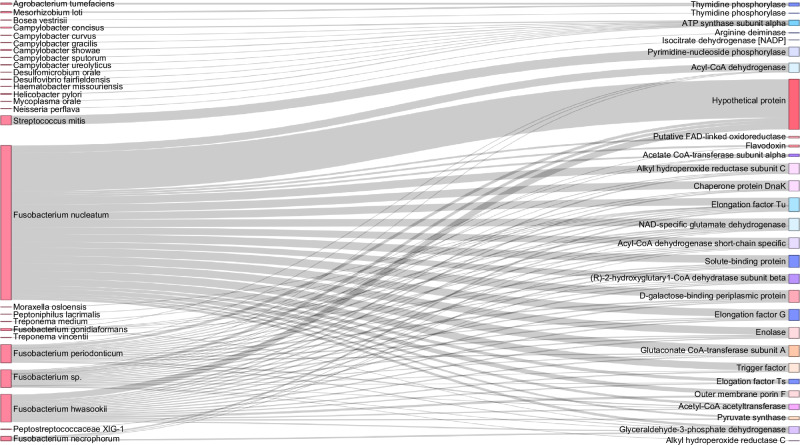
Distribution of proteins among bacterial species identified in OSCC secretome. Sankey diagram representing the protein distribution between bacterial species identified in oral cancer explants

The contribution of pathogens in the progression of oral cancer has been suggested by several-research groups^15,26,43^. We have previously identified pathways associated with Vitamin D-mediated Th2 responses in OSCC^44^, however we did not explore the potential pro-tumoral role of the microbiota. In this study, we analyzed the host and microbe proteomic landscape in the secretome of biopsies from patients with oral cancer compared to biopsies from healthy controls (Supplementary Table 1). Significantly upregulated pathways were revealed in human malignant samples such as response to organic substance/chemical, cell migration, intermediate filament cytoskeleton organization, cell adhesion, metabolism of amino acids and derivatives and infectious diseases (Fig. 1a). Interestingly, several downregulated pathways in oral cancer were associated with the regulation of the complement cascade (Fig. 1a). We then analyze activated upstream regulators by Ingenuity Pathways Analysis (IPA) and the data revealed the top 3 upstream regulators: MYC, lipopolysaccharide (LPS) and 1,2-dithiol-3-thione (D3T), suggesting that gram-negative bacteria could be an important modulator of cancer progression in OSCC (Fig. 1b). To evaluate the tumoral microbiome, bacterial peptides analyzed by mass spectrometry were identified with PEAKS X+ software and label free quantification was processed by PEAKSQ. We started with the analysis of the bacterial proteins in triplicate, obtaining 441 peptides in the healthy control samples (Fig. 1c) and 1 131 peptides in cancer samples (Fig. 1d). Between conditions, a total of 337 peptides were shared between oral cancer and healthy tissue secretomes, whereas 794 peptides were unique for oral cancer and 104 peptides were present only in the control condition (Supplementary Fig. 1A), with a different profile of differentially expressed peptides between healthy and malignant samples (Supplementary Fig. 1B). For the healthy tissue explant secretome (Control), we identified bacterial peptides corresponding to Malate dehydrogenase from *Aggregatibacter actinomycetemcomitans*, Triosephosphate isomerase from *Corynebacterium coyleae* and Glutathione reductase from *Listeria monocytogenes* (Fig. 1c, Supplementary Table 2). The bacterial peptides present in oral cancer explant secretomes belong mainly to *F. nucleatum* (Figs. 1d & 2, Supplementary Table 2). Thus, our results strongly suggest that under pathophysiological conditions of oral cancer, the primary bacterial colonization is likely attributed to *F. nucleatum*, implying that this bacterium may play a pro-tumoral role.

### Bacterial peptides from *F. nucleatum* are associated to the L-Glutamate degradation pathway

**Fig. 3:**
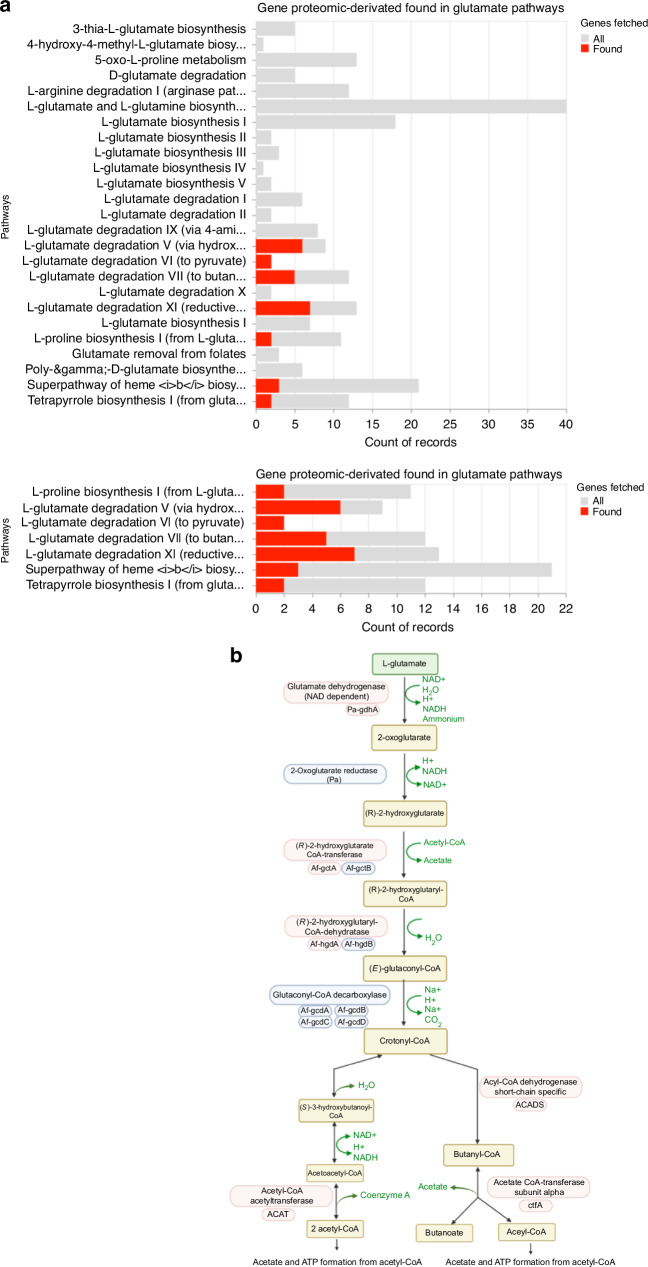
Metabolic pathways of L-Glutamate degradation are associated to *F. nucleatum* pepetides. **a** Bar chart of unsupervised and reproducible analysis of 25 metabolic pathways from MetaCyc containing 135 unique protein-encoding genes (in gray), from which the presence of protein-encoding genes derived from the oral malignant microbiome from *F. nucleatum* (in red) were present in metabolic pathways associated with L-Glutamate degradation, but not in L-Glutamate biosynthesis pathways. **b** Schematic representation of *F. nucleatum* enzymes found in the proteome of malignant samples (red) within the L-glutamate degradation pathway

**Fig. 4:**
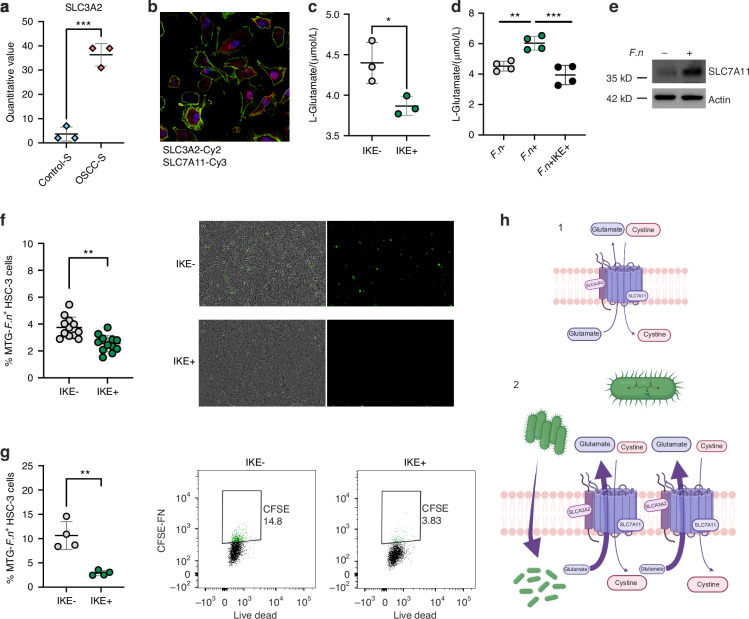
The L-Glutamate degradation pathway shows an association with bacterial peptides derived from *F. nucleatum*. **a** Scatter plots of quantitative value of SLC3A2 from the human proteomic dataset in Fig. 1. **b** Confocal image of the cystine/glutamate antiporter (System x_c_^-^) showing the expression and colocalization of SLC3A2 (green) and SLC7A11 (red) in HSC3 cells. **c** Scatter plots of extracellular L-glutamate from HSC3 cell cultures in the presence or absence of the Imidazole Ketone Erastin (IKE) and **d** in HSC3 cells uninfected or infected with *F. nucleatum* for 24 h. **e** Western blot of SLC7A11 in the HSC3 cells uninfected or infected with *F. nucleatum* for 24 h. **f** Scatter plots and representative images or dot plots of bacterial infection in the absence or presence of IKE by Incucyte and **g** Flow cytometry. **h** Schematic overview of *F. nucleatum* influencing the System xc- and L-Glutamate efflux. For all statistical analysis T test was used, ****P < 0.000 1, ***P < 0.001, **P < 0.01 and *P < 0.05 were considered significant

After identifying *F. nucleatum* as the primary bacterium in OSCC, we search for metabolic pathways associated with the specific bacterial peptides identified in OSCC samples. Interestingly, several enzymes from the L-Glutamate degradation pathway, highlighted in red, were present in malignant samples (Fig. 3a), suggesting that *F. nucleatum* catabolize L-Glutamate into butyrate (Fig. 3b). Since it has been shown that *F. nucleatum* metabolize L-Glutamate as a major source of energy^45,46^, we analyze the presence of glutamate transporters in the host proteomic dataset. In this context, our proteomic results revealed that one of the main overexpressed targets was the chaperone protein SLC3A2 (Fig. 4a). Interestingly, the cystine/glutamate antiporter (SLC7A11) forms a complex with the chaperone SLC3A2, known as System x_c_^-^^47^. Thus, we measured and confirmed the expression and colocalization of SLC7A11 and SLC3A2 in the OSCC cell line HSC3 (Fig. 4b). Therefore, we assessed whether the System x_c_^-^ is functional for glutamate efflux in the HSC3 cell line. Our findings revealed that HSC3 cells release glutamate into the extracellular medium in a x_c_^-^ dependent manner, as the use of imidazole ketone erastin (IKE), a potent and selective inhibitor of System x_c_^-^^48^, significantly reduced L-glutamate efflux (Fig. 4c). We confirmed that IKE inhibited the expression of SLC7A11 without affecting cell survival in HSC3 cells (Supplementary Fig. 2). Then, we analyzed the effect of *F. nucleatum* in the x_c_^-^ System and we observed that the presence of the bacterium was promoting the L-Glutamate efflux, an effect selectively inhibited by IKE (Fig. 4d), suggesting that *F. nucleatum* induces L-Glutamate efflux via the System x_c_^-^. At the same time, our data showed that the presence of *F. nucleatum* promotes the overexpression of SLC7A11 in HSC3 cells (Fig. 4e). Finally, *F. nucleatum* infection was evaluated in the presence of IKE and we observed that the inhibition of the System x_c_^-^ reduced the infection capacity of *F. nucleatum* by using IncuCyte® Live-Cell Analysis System (Fig. 4f) and flow cytometry (Fig. 4g) in live HSC3 cells. In summary, our data suggest that the System x_c_^-^ (SLC7A11 and SLC3A2) (Fig. 4h.1) is exacerbated by the presence of *F. nucleatum*, promoting more L-Glutamate efflux and bacterial infection (Fig. 4h.2).

### Tumoral growth and OSCC cells migration is promoted by *F. nucleatum* infection

**Fig. 5:**
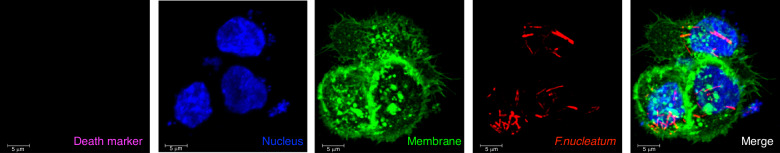
Confocal images of Oral cancer cells after 6-hour infection with *F. nucleatum.* Confocal images of HSC3 cells infected with *F. nucleatum* (6 h). Bacteria was stained with CSFE (red) previous the infection. HSC3 were stained with Hoechst (blue), plasma membrane cell mask deep red (green) and propidium iodide (magenta) to confirm bacteria viability

**Fig. 6:**
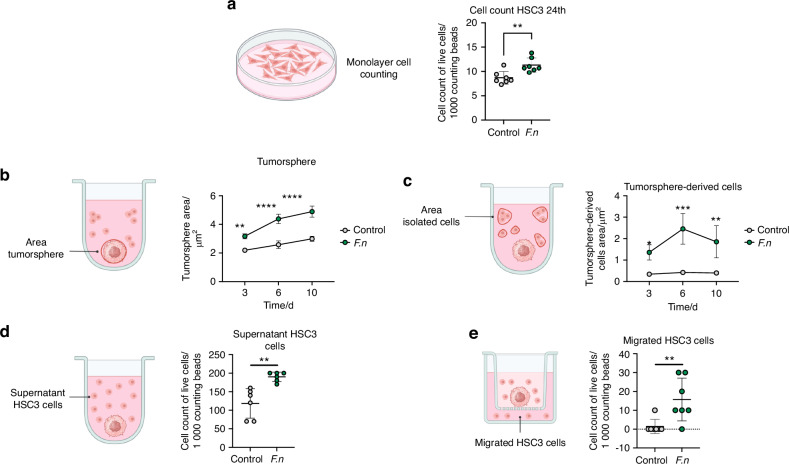
*F. nucleatum* infection drives tumoral growth and enhances the migratory behavior of OSCC cells. **a** Representative schema and scatter plot of cell counting of uninfected *or F. nucleatum*-infected HSC3 cells in monolayer (24 h). **b** Representative schema and point-&-connection line plot of tumourspheres area of uninfected or *F. nucleatum*-infected HSC3 cells at day 3, 6 and 10. **c** Representative schema and point-&-connection line plot of isolated cells from tumorsphere of uninfected or *F. nucleatum*-infected HSC3 cells at day 3, 6 and 10. **d** Representative schema and scatter plot of isolated HSC3 cells in supernatants from tumourspheres of uninfected or *F. nucleatum*-infected HSC3 cells after 6 days of infection. **e** Representative schema and scatter plot of isolated HSC3 cells in supernatants from de lower chamber of transwell from tumourspheres of uninfected or *F. nucleatum*-infected HSC3 cells after 6 days of infection. Data are presented as individual symbols with paired lines (Paired t test). For statistical analysis, Two way ANOVA and T test were used,****P < 0.000 1, ***P < 0.001, **P < 0.01 and *P < 0.05 were considered significant

According to the host pathways observed in Fig. 1 and the infection revealed in Fig. 3, 4, we evaluated whether *F. nucleatum* affect cell migration after HSC3 infection. The internalization of *F. nucleatum* into the HSC3 cell was confirmed by live-cell confocal images, after 6 h of culture. *F. nucleatum* was observed inside live cancer cells at 6 h postinfection, where propidium iodide (magenta label) was used to detect dead bacteria or dead HSC3 cells, thus bacterial infection did not induce cancer cell death (Fig. 5). To discard the possibility that *F. nucleatum* was attached to the cell surface and not inside of HSC3, cells were cultured in the presence of antibiotic with broad-spectrum anaerobic activity, metronidazole and gentamicin, to kill bacteria outside the cells after infection. In addition, we confirmed *F. nucleatum* entrance to HSC3 cells by 3D confocal images and video (Supplementary Fig. 3). Interestingly, at longer infection time points (48 h), OSCC cells started to detach (data not shown). The effect of *F. nucleatum* on tumoral growth was evaluated by 2D (monolayer) and 3D (tumorsphere) model of oral cancer. First, we performed a monolayer infection assay for 24 h, where the cell counting revealed a significant increase of *F. nucleatum*-infected HSC3 cells in comparison with non-infected cells (Fig. 6a). In accordance with the increased cell counts in infected cells, we evaluate the effect of the bacteria regarding tumorsphere formation and cell detachment from the 3D tumorsphere (Supplementary Fig. 4). Our results revealed that the area of tumorspheres from infected cells shown in Supplementary Fig. 4 was significantly larger than the tumorsphere from non-infected cells (Fig. 6b). Moreover, the area of isolated cells detached from spheroids in infected tumourspheres was higher in comparison with non-infected tumourspheres (Fig. 6c), thus *F. nucleatum* not only increased spheroids size over time, but also promoted cancer cell migration. To confirm the effect of *F. nucleatum* on cell migration, cell counting was evaluated in the supernatant of tumourspheres and by transwell assay (Fig. 6d, e). In both cases, this periodontal bacterium significantly increased the number of cells in the supernatants surrounding the tumorspheres (Fig. 6d) and through transwell in vitro migration (Fig. 6e). These results suggest that *F. nucleatum* infection in OSCC promotes a more aggressive phenotype of this type of cancer, enhancing tumor growth and increasing migration.

### *F. nucleatum* induces EMT markers expression on OSCC cells

**Fig. 7:**
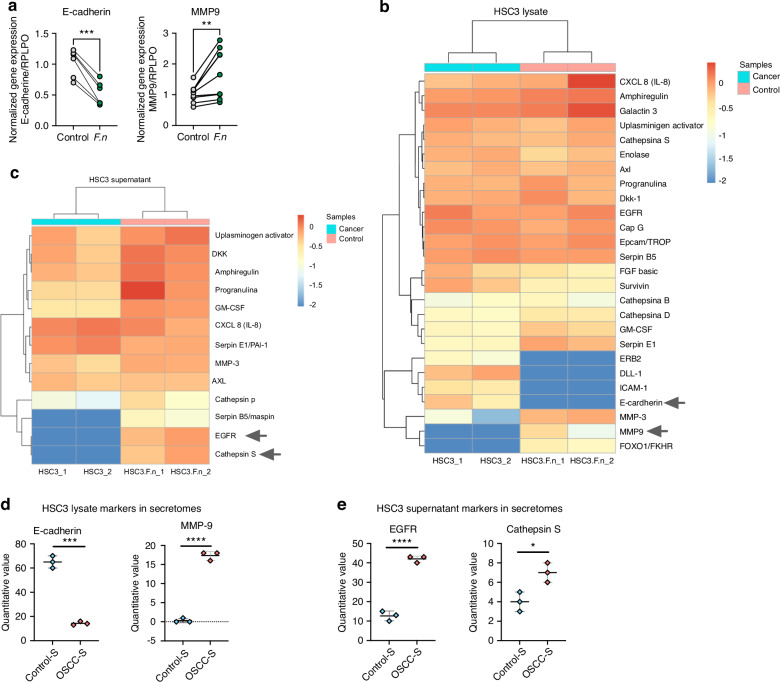
*F. nucleatum* promotes ETM markers expression in OSCC. **a** Symbols and line plots of E-cadherin and MMP9 mRNA expression uninfected or *F*. *nucleatum*-infected HSC3 cells in monolayer (6 h). Data are presented as individual symbols with paired lines (Paired t test). **b** Differential protein expression heatmap of EMT markers from lysate of uninfected or *F. nucleatum*-infected HSC3 cells in monolayer (24 h). **c** Differential protein expression heatmap of EMT markers from supernatants of uninfected or *F. nucleatum*-infected HSC3 cells (24 h). **d, e** Scatter plots of quantitative value of E-cadherin, MMP9, EGFR and Cathepsin S from the human proteomic dataset in Fig. 1. For statistical analysis, Sidaks multiple comparisons and T test were used, ****P < 0.000 1, ***P < 0.001, **P < 0.01 and *P < 0.05 were considered significant

As we observed a potential EMT behavior in OSCC cells after *F. nucleatum* infection and based on the increased tumor size and cell migration, we evaluated the expression of E-cadherin (EMT markers) and MPMP-9 in HSC3 cells after 6 h of *F. nucleatum* infection. The mRNA MMP-9 expression was significantly higher on OSCC cells after *F. nucleatum* infection and mRNA E-cadherin expression significantly decreased after the co-culture with the bacteria (Fig. 7a). To further investigate EMT the acquisition of the mesenchymal phenotype, we performed a Proteome array for cancer proteins. *Human XL Oncology Array Kit* exhibited 26 differentially expressed proteins in the cellular lysate and 13 differentially expressed proteins in the supernatant of HSC3 cells after 24 h of *F. nucleatum* infection (Supplementary Fig. 5A). FOXO1/FKHR, MMP-3, MMP-9 and SerpinE1 were significantly higher expressed on HSC3 cellular lysate, after *F. nucleatum* infection, whereas survivin, ERB2, ICAM-1, DLL-1 and E-cadherin significantly decreased after *F. nucleatum* infection (Fig. 7b & Supplementary Fig. 5B). Proteins from supernatants from infected and non-infected OSCC cell cultures were also evaluated with the *Human XL Oncology Array Kit* (Fig. 7c). Progranulin, GM-CSF, Cathepsin S, Serpin B5/Maspin and EGFR significantly increased their expression on supernatants from HSC3 infected cells (Fig. 7c). Finally, we evaluated the presence of EMT proteins in the human secretome dataset obtained from OSCC and control tissues and we observed significant downregulation of epithelial marker E-cadherin and an upregulation of mesenchymal markers MMP-9 (Fig. 7d). In addition, Cathepsin S and EGFR were also upregulated in cancer secretomes in comparison to control samples (Fig. 7e). Collectively, these findings provide further support the hypothesis that *F. nucleatum* infection in OSCC contributes to a more aggressive phenotype by inducing EMT.

### Butyrate stimulates tumor growth in a dose-dependent manner in OSCC tumorspheres

**Fig. 8:**
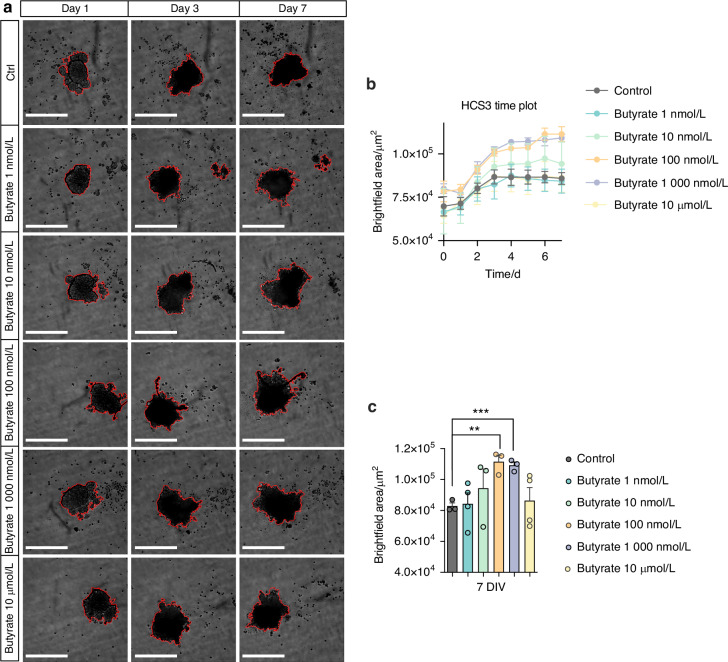
Butyrate intermediate concentrations encourage HSC3 tumorsphere growth. **a** Representative images by live cell microscopy using the IncuCyte system to determine HSC3 tumorsphere growth, after being challenged with different concentrations of butyrate. **b** Time plot of HSC3 tumorsphere size after butyrate treatment for 7 days. **c** HSC3 tumorsphere size after butyrate treatment at day 7. Data represent the mean ± SEM of at least 3 independent biological replicates. Data represent the mean ± SEM of at least 3 independent biological replicates. t test student, **P < 0.01; ***P < 0.001

Butyrate was the main metabolite derived from the L-Glutamate degradation pathway (Fig. 3b) and it has been associated with cancer progression^49^. In this scenario, the effect of butyrate on OSCC tumorspheres growth was evaluated by challenging them with increasing concentrations of butyrate (Fig. 8a). Kinetic graphic showed that butyrate stimulates tumorsphere growth at day 7 using 100 and 1 000 nmol/L of butyrate (Fig. 8b, c), however, the highest concentration tested (10 μmol/L) tested did not change tumorsphere size (Fig. 8b, c). Thus, it is tempting to speculate that *F. nucleatum* may incorporate the glutamate released from OSCC tumor cells, metabolize it into butyrate, which could subsequently be utilized by tumor cells to promote their growth.

### Increased GAL-9 on OSCC-infected cells

**Fig. 9:**
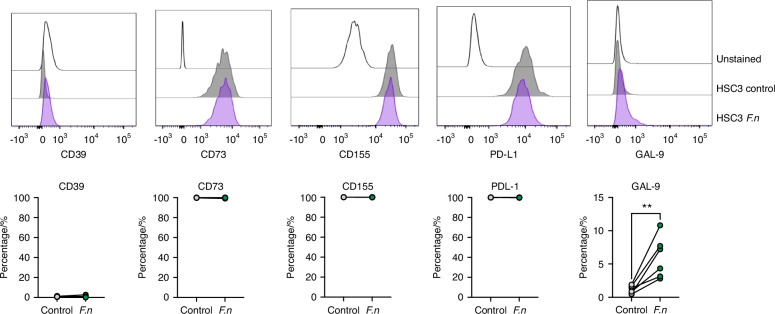
GAL-9 is induced on OSCC cells after *F. nucleatum* infection. Histograms and total percentage of cells expressing CD39, CD73, CD155, PDL-1 and GAL-9 from uninfected or *F. nucleatum*-infected HSC3 cells (24 h). For all statistical analysis, T test was used, ****P < 0.000 1, ***P < 0.001, **P < 0.01 and *P < 0.05 were considered significant

OSSC behavior is impacted by the presence of immunosuppressive molecules, especially on the response of tumors to immunotherapy, chemotherapy, and radiotherapy^50^. CD39, CD73, Galactine 9, CD155 and PDL-1 has been described as immunosuppressive molecules in OSCC cells^37,39,40,42,51^. In this context, we evaluated the expression of these molecules after 24 h of *F. nucleatum* infection. We noticed that CD39 is not expressed by HSC3 cells whereas CD73, CD155 and PDL-1 are fully expressed by these cancer cells, and their expression did not change with *F. nucleatum* infection (Fig. 9). Interestingly, Gal-9 showed differential expression after *F. nucleatum* infection. In fact, the periodontal bacteria significantly increased Gal-9 in OSCC cells (Fig. 9).

### Complement proteins in oral cancer

**Fig. 10:**
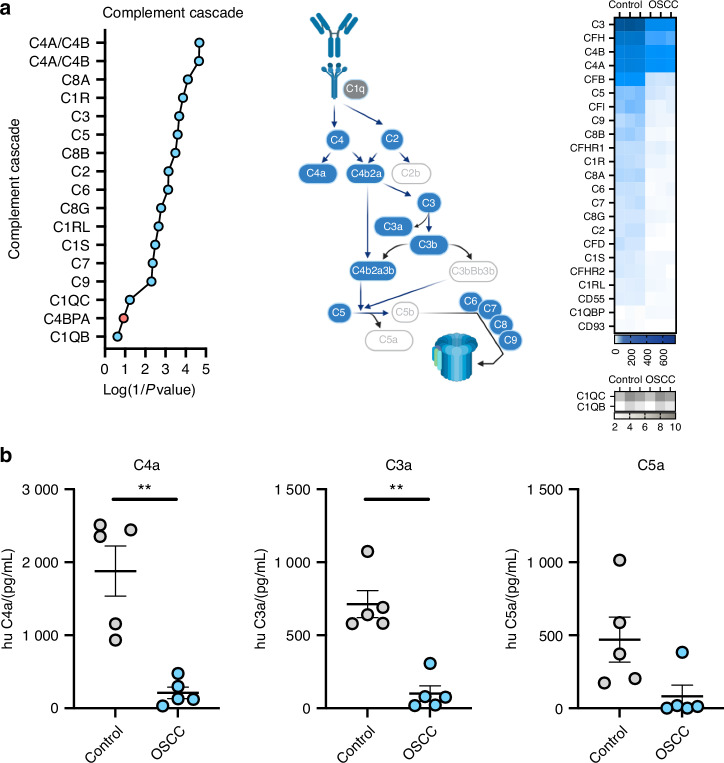
Complement cascade proteins identified on OSCC secretome. **a** Plots, schematic representation, and differential protein expression heatmap of proteins from the complement cascade protein from the proteomic dataset in Fig. 1. **b** Scatter plots of soluble C4a, C3a and C5a in control and OSCC secretome. For all statistical analysis, T test was used, ****P < 0.000 1, ***P < 0.001,**P < 0.01 and *P < 0.05 were considered significant

Since the complement system was downregulated in the host proteomics and this cascade has been associated with innate responses against pathogens^52,53^, we evaluated the main complement proteins from the human secretome dataset and we observed that most complement proteins in OSCC samples were reduced in comparison with the control samples (Fig. 10a). Complement proteins were then measured in the secretome to validate the differences observed in the proteomic data. Our data showed that complement proteins C3a and C4a were reduced in OSCC secretome compared to the control secretome (Fig. 10b). Overall, our data showed a potential dysregulation of the complement cascade in cancer samples. Further studies are required to understand the dual role of these soluble factors between tumor progression and the antitumor immune response.

## Discussion

Oral microbiome imbalance of susceptible individuals can lead to the development of both oral and systemic diseases. In this study we identified the host-pathogen pathways from the secretome of OSCC biopsies compared with healthy biopsies. Markedly increased activity in pathways related to responses to LPS, cell migration, organization of intermediate filament cytoskeleton, cell adhesion and amino acid and derivative metabolism were detected in human malignant samples, and interestingly, pathways associated with the regulation of the complement cascade were found downregulated in oral cancer. Microbiome proteomics results reveled that *F. nucleatum* has the higher presence of peptides on OSCC secretome compared with control and notably, various enzymes associated with the L-Glutamate degradation pathway were detected in the OSCC samples, indicating the involvement of *F. nucleatum* in the catabolize of L-Glutamate into butyrate. Also, we observed that bacterium promote the L-Glutamate efflux from HSC3 by the System x_c_^-^. As proteins associated with cancer growth and EMT markers were identified on OSCC secretome, we demonstrated in vitro, the protumoral mechanisms of *F. nucleatum* in an OSCC cell line.

From the last past decade, numerous scientific findings have illustrated the changed bacterial colonization in cancerous tissue. Hooper et al., detected for the first time viable bacteria within the tissue of oral squamous cell carcinoma^19,54^ and with emergence of NGS, several studies have assessed to bacterial profiles associated with OSCC^15–18^. However, recent research has focused on identifying the specific bacteria and their mechanism participating on OSCC development and progression. According to our analysis, *F. nucleatum* was the main bacteria detected in malignant tissues. This bacterium has been recognized as an oncogenic bacteria, especially on colorectal cancer, according to its virulence factors^24^. For example, the fusobacterial adhesin (FadA) is one of the main virulence factor from *F. nucleatum*^55^ and its binding to E-cadherin in cancer cells activates b-catenin signaling and regulates the inflammatory and oncogenic reactions^25^. In addition, FadA activate cadherin/β-catenin pathway of cancer cells, leading to upregulation of chk2, a checkpoint enzyme, responsible of DNA damage^27,56^. Based on our results and previous studies, *F. nucleatum* participates on OSCC progression through several mechanism. Recently, Kamarajan et al., reported that *F. nucleatum* enhanced tumorsphere formation and cell migration/invasion via integrin/TLR pathway and Nisin, an antibacterial peptide produced by commensal bacteria^26^. Our resulted showed cancer cell migration and tumorphere formation promoted by the bacteria, but we also observed higher cell detachment from the tumorsphere associated to *F. nucleatum* infection. Likewise, butyrate, a derivates metabolic product of the bacteria, also modulated tumorphere formation. Butyrate is produced by periodontal pathogens fermentation in the oral cavity and it has been demonstrated to contribute to the occurrence and development of periodontitis^57^. Its role in cancer is controversial, some studies postulated antitumoral effects^58^, based on its great efficiency in maintaining intestinal epithelial barrier function and regulating host mucosal immune responses^59^, however, other researchers have elucidated protumoral effects on OSCC. According to Zang et al., butyrate could promoted cell migration, invasion and the expression of EMT markers such as SNAI1 and Vimentin in oral cancer cells^49^. We observed both effects associated to butyrate concentrations, intermediate concentrations stimulate tumorsphere growth, but higher concentrations did not affect the tumorsphere size.

Indeed, the most associated protumoral mechanism of *F. nucleatum* is EMT. EMT, or epithelial-mesenchymal transition, is a dynamic and reversible process in which epithelial cells undergo a transformation into a mesenchymal cell phenotype. This transformation is marked by a notable increase in the production of extracellular matrix (ECM) components, enhanced migratory capability, increased invasiveness, and a resistance to apoptosis^60^. Our findings suggested the acquisition of this phenotype by the oral cancer cells, after *F. nucleatum* infection. This phenomenon has been studied by several researchers in both oral and colorectal cancer^30,61^ principally by the expression of classical markers on cancer cells, after the infection. Harrandah et al., reported increased expression of mesenchymal markers such as MMP-9 and MMP-1 after poli infections, nevertheless showed that *F. nucleatum* alone had comparable or greater effects than the four bacteria together^29^. Our data revealed increased levels of MMP-9 on infected cancer cells, but also decreased levels of E-cadherin after the infection, which is described as a key event of EMT, permitting the separation of individual cells from the primary tumor mass, and therefore promoting cancer invasion^62^.

On another hand, it is well characterized that *F. nucleatum* can induce significant changes in the expression of genes related to immune defense responses during periodontal diseases^63^. Moreover, according to prior reports, *F. nucleatum* modulates the local immunity of cancers by creating a permissive tumor microenvironment, insensitive to pro inflammatory signals, with low TLR4 signaling and recruitment of type 2 Macrophages^64^. Our in vitro results illustrated differential expression of immunosuppressive markers on oral cancer cells after *F. nucleatum* infection. Gal-9 was highly expressed on infected cells; it has been previous identified on OSCC lesions and it has been proposed as an important marker on differential diagnosis between oral squamous cell carcinoma and other oral lesions^65^. It has immunoregulatory properties, inducing Treg expression via TGF-β and Th1 apoptosis by Tim-3/Gal-9 pathway^38,66^. Furthermore, we identified significantly decreased expression of DLL-1, which is part of Notch/DLL-1 pathway associated to enhance T cell infiltration into tumors and elevated numbers of CD44^+^CD62L^+^CD8^+^ memory T cells^67^. The differential expression of these molecules after *F. nucleatum* infection could alter antitumoral immune responses and indeed enhance tumoral growth and progression.

Since *F. nucleatum* was the main specie detected in the secretome of OSCC, the tumor-colonization skills of this bacteria were another important aspect to evaluate. The mechanisms that allow its colonization are still unclear, however, according to our findings, we proposed that is through its metabolic pathways. *F. nucleatum* appears to be one of the few anaerobic bacteria that can obtain energy also from amino acids. Indeed, it can survive by obtaining energy only from the degradation of L-glutamate^46,68^. Surprisingly, several enzymes from the L-Glutamate degradation pathway were identified in the OSCC samples and thus we confirmed that HSC3 was able to efflux L-Glutamate to the extracellular by the System x_c_^-^. Previous studies have reported the expression of this antiporter and glutamate receptors on oral cancer cells^69,70^, however, we evaluated for the first time the link of the periodontal bacteria and the System x_c_^-^. It seems that the System x_c_^-^ plays and important role during *F. nucleatum* infection in cancer cells, because the bacterium significantly promoted L-Glutamate efflux from HSC3 and the expression of SLC7A11 in HSC3 cells, but when an inhibitor of this antiporter was applied, the effects were reverted. Based in our data, we believe that *F. nucleatum* take advantage of glutamate to colonize and infect OSCC cells, promoting the expression of the System x_c_^-^ to enhance the glutamate efflux, allowing more colonization in a positive feedback manner.

Additionally, the secretome analysis revealed a potential dysregulation in the complement cascade in OSCC, illustrated by reduce levels of proteins C3a and C4a. The complement cascade plays an important role in the innate response against bacterial infection and in the recruitment and regulation of inflammatory cells, participating in the pathogenesis of periodontitis^52^. The reduced levels of complement proteins could also favor bacteria colonization into OSCC cells.

This study has the following limitations. First, in vivo studies are required to confirm *F. nucleatum* protumoral mechanisms identified in our in vitro data. Second, it is well known that the oral microbiome is a complex ecosystem. The interactions of *F. nucleatum* with other bacteria/microorganisms and the existence of other strains present in the tumor environment were not explored in depth. Third, *F. nucleatum* was not measured in the periodontal plaque of the patients.

Taken together this evidence, oral dysbiosis and the presence of specific bacteria in the tumor environment seems to play an important role in the prognosis of prevalent malignant diseases, so it is crucial to establish preventive measures associated with periodontal health care, both in healthy and OSCC patients, and thus contribute to a better prognosis, response to treatment and therefore a better quality of life for them.

### Methodology

#### Patient data

Patients with and without OSCC were selected and consented in accordance with the Talcahuano Health Service Research Ethics Committee, reference number 19-06-11 and Concepcion Health Service Research Ethics Committee, reference number 19-03-07 and in accordance with the Declaration of Helsinki. Patient data is summarized in Supplementary Table 1. OSCC and healthy biopsies were obtained from patients, after informed consent was approved.

#### Sample collection and processing

The biopsies obtained were transported to the laboratory in 10 mL of serum-free X-VIVO15 medium (LONZA) at 4 °C and processed in a time course of 2-3 h. For secretome collection, a piece of tissue (weight approximately 0.1 g) was cut from the oral cancer and control biopsies and incubated in serum-free X-VIVO15 (LONZA) medium for 48 h at 37 °C. After incubation, the medium was collected, debris was removed by centrifugation and filtration (0.22 μm), and the medium with all proteins and factors secreted from the tissue (Secretome) was stored at −80 °C until use. Data were obtained from a triplicate analysis of 2 conditions: 500 μL of Secretome “OSCC” and 500 μl of Secretome “Healthy Control”. Each of these two conditions consists of a pool of secretome samples (100 μL each) from 5 OSCC patients and 5 control individuals. In addition, an X-VIVO15 medium condition was analyzed only in order to rule out potential contaminants from the medium, since this medium was used for secretome collection. Once the samples were selected and both pools were generated, protease inhibitor and 1X phosphatase were added. The samples were cold homogenized using ultrasound and centrifuged to remove debris. Subsequently, the proteins contained in the samples were precipitated using cold acetone overnight at −20 °C. The proteins obtained were dried in a rotary concentrator at 4 °C. The samples were resuspended in urea and bicarbonate, reduced with dithiothreitol (DTT), alkylated with iodine acetamide and subsequently diluted with ammonium bicarbonate. The samples were digested with Trypsin/Lys-C overnight at 37 °C and the peptides obtained were dried in a rotary concentrator at 4 °C and resuspended in formic acid. Finally, 200 ng of the peptides obtained from condition 1) Secretome “OSCC” 2) Secretome “Healthy Control” and 3) X-VIVO15 medium were injected in triplicate into a timsTOF Pro mass spectrometer (Bruker Daltonics). The collection of results was performed under the use of oTOF software (Bruker Daltonics).

The mass spectrometry human proteomics data have been deposited to the ProteomeXchange Consortium via the PRIDE partner repository with the dataset identifier PXD023049.

#### Human functional enrichment analysis

Two statistical analyzes were performed associated with the genes derived from the proteomics analyses: i) Ingenuity Pathways Analysis (IPA), performed with the tool QIAGEN IPA, ii) Gene Ontology Enrichment Analysis (GOEA) performed with the g-profiler platform, https://biit.cs.ut.ee/gprofiler/gost^71^. The raw data from both analyzes were processed in Python and graphs were developed for interpretation^72^. The code is available at https://github.com/Nanocell-Lab/metabcrosstalk.

#### Bacterial automatics glutamate pathway search in Metacyc

The identification of genes derived from the proteomics analyzes in the metabolic pathways involved in L-glutamate was automated. A code was developed in python that consists of three steps: i) identification of the genes derived from the proteins identified in the proteomic profiles using the database of HOMD^73^, ii) identification of the metabolic pathways in Metacyc^74^ associated with a metabolite, and iii) counting of the genes found in each metabolic pathway. The source code is available at https://github.com/Nanocell-Lab/metabcrosstalk.

#### Protein identification

Data analysis was performed by PEAKS Studio version X+ software (Bioinformatics Solutions Inc., Waterloo, Canada). All annotated protein sequences of genomes from the expanded Human Oral Microbiome Database (eHOMD, www.homd.org/) were used as a database (5.041.813 sequences, accessed 19/08/2021). The parameters used were 50 ppm as mass tolerance using monoisotopic masses and ionic fragments of 0.05 Da. Trypsin was used as digestion enzyme, specific digestion mode, and a maximum of 2 missed cleavages per peptide. Carbamidomethylation (fixed PTM) of cysteine, Oxidation of Methionine, Acetylation of Lysine, Deamination of Asparagine and Glutamine and Carbamylation of Lysine and Nterminal were used as PTM. In the same way, the culture medium used in the experiment was analyzed, which allowed discarding the proteins detected in the secretome runs. Additionally, FDR estimation was included by means of a decoy database. The filters used were an FDR ≥ 1% and at least 1 unique peptide per protein.

#### Protein quantification LFQ “Free Label Quantification”

Quantification was performed using the LFQ (“Label free Quantification”) strategy, using PEAKS Studio X+ software (Bioinformatics Solutions) with the PEAKSQ quantification module. As parameters at least one peptide was set to make pairwise comparisons between two random samples of the assay, retention time of 20 min, ion mobility (1/K0) with tolerance 0.05, normalization using TIC (“Total Ion Current”), FDR of 1% and a fold change of 1.

#### Cell culture of oral squamous cell carcinoma cancer HSC3

Oral squamous cell carcinoma cancer HSC3 (Merck, Cat. # SCC193) cells were kindly donated by Dr. Wilfredo Gonzalez from Universidad de Los Andes (Santiago, Chile). These cancer cells were maintained in DMEM/GIBCO supplemented with 10% fetal bovine serum (SV30160.03, HyClone cytiva, collected in Paraguay, processed in France), penicillin/streptomycin (1:100) (15140-122 Gibco®, Carlsbad, CA, USA), and glutamina/glutamax (1:100) (10378-016 Gibco®, Carlsbad, CA, USA) incubated at 37 °C, 5% CO_2_.

#### Bacterial culture

*F. nucleatum* strain ATCC 25586 were grown in anaerobe basal broth (CM0972, OXOID) at 37 °C in an anaerobic chamber according to manufacture instructions. For infection assay bacteria were grown until their exponential growth phase (0,35 OD), with growth rate measured by optical density at 600 nm.

#### Infection assay

HSC3 cells (1 × 10^6^) were seeding in tubes, incubating at 37 °C in 5% CO_2_. Bacteria were grown, suspended in DMEM media, and added to a multiplicity of infection (MOI) of 100 approximately. The tubes were centrifuged a 300 × *g* for 20 min to ensure the contact between the cell layer and the bacteria; after centrifugation, tubes were incubated for 90 min at 37 °C in 5% CO_2_ to allow for internalization of bacteria^75^. Cells were then washed and incubated with fresh medium (DMEM) supplemented with gentamicin (G1914 Sigma-Aldrich®, Gillingham, UK) and metronidazole (M3761 Sigma-Aldrich®, Gillingham, UK), (300 µg/mL and 200 µg/mL respectively) for the postinfection times defined.

#### Super resolution live cell microscopy

*F. nucleatum* infection on HSC3 cells was observed by confocal images. Before the infection, the bacteria was stained with *CFSE CellTrace™* (green dye Thermo Fisher). After every time of infection 1 × 10^4^ HSC3 cells were seeded in cell view cell culture slide, glass bottom, advanced TC (Greiner Bio-One #543979) and stained with Hoechst 33342 (Invitrogen) (0.1 μg/mL) for nucleus and cellmask Deep red (0.3X, Invitrogen) for plasma membrane. Cancer cells were stained with propidium iodide (1X. Invitrogen) to confirm bacteria/cells viability only at 6 h of infection. Live cell images were taken 6-, 24- and 48-hour after infection assay by confocal microscope *Leica Sp8* with super-resolution module per lighting, at 37 °C and 5% CO_2_. The HC PL APO 63x/1,40 OIL CS2 oil immersion objective was used. The images were acquired in *x,y,z* at a size of 1 024 × 1 024 and are represented as projection of maximum intensity generated in the LASX software.

#### Immunocytochemistry

6 × 10^4^ HSC3 cells were seeded onto glass coverslips for 24 h. Subsequently, the cells were fixed with 4% PFA for 30 min. Next, three washes were performed with 1X PBS, followed by overnight incubation with primary antibodies against SLC3A2 (Santa Cruz #SC-59145) and SLC7A11 (Cell Signaling #12691). The following day, cells were washed three times with 1X PBS and incubated with Hoechst 33342 as a nuclear marker and secondary antibodies Anti-mouse Cy2 and anti-rabbit Cy3 (The Jackson Laboratory) for 2 h. Finally, samples were washed with 1X PBS and mounted on slides using a fluorescent mounting medium (DAKO). Images were acquired using the Leica SP8 super-resolution confocal microscope. The HC PL APO 63x/1.40 OIL CS2 oil immersion objective was utilized. Images were acquired in x,y,z at a size of 1 024 × 1 024 and represented as maximum intensity projections generated in the LASX software.

#### Cell counting

HSC3 cells were counted after 24 h of infection, using CountBright Absolute Counting Beads, stained with Live/Dead dye (L34974, Invitrogen, Thermo Fisher Scientific) and analyzed by flow cytometry.

#### Tumorsphere formation

A tumorsphere is defined as an aggregate of cells that is at least 50 μm in diameter. HSC3 cells were challenged with *F. nucleatum* for 90 min, then tumorspheres were developed by maintaining cells under suspension on ultra-low cluster polystyrene plates (ULA #7007) and centrifugating a 400 g for 10 min. After 3 days the tumorsphere formation was confirmed microscopically. The total area occupied by tumorspheres and isolated cells in each well was measured by *Motic AE31* microscopy, ProgRes®MF^cool^ and Image J software.

#### Transwell assays

These assays were carried out using Transwell chambers (5 μm pore size; Corning). Tumor spheres from infected/noninfected cancer cells were suspended in 100 μL DMEM medium, after 4 days of infection. 100 μL of DMEM medium was added in the lower chamber. After 2 days, cells from the supernatant of the tumoursphere and cells migrated to the lower chamber were collected, counted using *CountBright Absolute Counting Beads*, stained with Live/Dead dye (Life Technologies) and analyzed by flow cytometry.

#### RT-qPCR

Total RNA from harvested cells was isolated using Trizol Reagent (Invitrogen®) and Direct-zol^TM^ RNA Microprep*, (*ZYMO RESEARCH) kit. The mRNA expression of the two markers (MMP9 and E-cadherine) was measured using quantitative real-time polymerase chain reaction (qPCR). The primers were previously designed in the Primer-BLAST (NCBH-NIH) platform and Ensembl Genome, using the sequences; MMP9 (F’ GCCACTACTGTGCCTTTGAGTC; R’ CCCTCAGAGAATCGCCAGTACT) and E-cadherine (F’ GTCTGTCATGGAAGGTGCT; R’ TACGACGTTAGCCTCGTTC). RPLPO expression levels were used as a normalizing endogenous control. The results were graphed using 2^-ΔΔCt^ method.

#### Proteome profile array

Cellular lysate and supernatant of HSC3 infected/noninfected cells was analyzed to determinate the expression of 84 proteins related to cancer. “*Proteome profile Array*^*TM*^*Human XL Oncology Array Kit”* (R&D Systems) was performed according to manufacturer’s instructions.

#### Flow cytometry

HSC3 cells after 24 h of infection were stained with anti-CD155(0.25 μg/mL), anti-Galg 9(100 μg/mL), anti-PDL-1(100 μg/mL), anti-CD73(200 μg/mL) and anti-CD39(100 μg/mL) (all BioLegend) for 30 min at 4 °C in the dark. Samples were acquired on LSR Fortessa (BD), and files analyzed using FlowJo (Tree Star). Gates were set based on biological controls and fluorescence minus one control (FMO).

#### Western blot for cystine/glutamate antiporter (SLC7A11)

The HSC3 cells were infected with *F. nucleatum* for 24 h. Subsequently, they were lysed with RIPA buffer supplemented with a cocktail of protease/phosphatase inhibitors (Cell Signaling #5872). Proteins were then extracted by centrifugation at 15 000 r/min for 15 min at 4 °C. From the supernatant, the total protein concentration was determined using the Bradford method. 30 μg of protein were loaded and separated on TGX FastCast Acrylamide 10% gels (BioRad #1610173). The proteins were then transferred to a PVDF membrane and incubated with anti-SLC7A11 antibody 1:1 000 (Cell Signaling #D2M7A) overnight. The next day, the membrane was incubated with an HRP secondary antibody (1:5 000) and the reaction was developed using the Western Lighting® Plus-ECL enhanced chemiluminescence substrate (PerkinElmer #NEL103001EA). Anti-actin (Santa Cruz #sc-47778) conjugated to HRP was used at 1:20 000 and incubated for 2 h at room temperature.

#### Inhibition assay of SLC7A11 mediated by imidazole ketone erastin (IKE)

1 × 10^4^ HSC3 cells per well were incubated on DMEM media supplemented with 10% fetal bovine serum, streptomycin (1:100), and penicillin (1:100) at 37 °C, 5% CO_2_. Before the infection, *F. nucleatum*, was stained with *CFSE CellTrace™* (green dye) and cancer cells media was changed to DMEM without antibiotics supplementation, then the bacteria was added to cancer cells at a multiplicity of infection (MOI). After 12 h of infection, IKE (MedChem #HY-114481) 5 µmol/L was added to infected and non-infected cells to blockade glutamate efflux. Bacteria colonization on HSC3 cells was measured by IncuCyte S3 and Flow cytometer after 24 h of glutamate blocking.

#### Butyrate assay

Tumorsphere from HSC3 cells were incubated in IncuCyte S3 for 3 days. Subsequently, a dose-response curve of butyrate was performed (1 nmol/L, 10 nmol/L, 100 nmol/L, 1 000 nmol/L, and 10 μmol/L). Kinetic images by live cell microscopy were obtained by IncuCyte S3 system every 4 h for 7 days to determine the growth of the tumorsphere.

#### Anaphylatoxin detection

Levels of complement proteins in cancer and control secretomes were determined by BD Cytometric Bead Array Human Anaphylatoxin Kit (BD) according to manufacturer’s instructions.

#### Statistical analysis

Statistical tests were performed using Prism 9 software (GraphPad). The analysis of graphical comparisons between 2 variables was carried out by T-test with paired and unpaired data, or in the case of more than 2 variables by ANOVA and Sidak´s multiple comparisons test. p values are reported as follows: *P < 0.05, **P < 0.01, ***P < 0.001, and ****P < 0.000 1.

In addition, after preprocessing proteomic data, we transformed it to a structure data by using R. To observe hierarchical data structure suitable for heatmap visualization using heatmaply. Besides, we used the circlepackeR function to generate circular layouts, facilitating the visualization of hierarchical relationships among proteins.

## Supplementary information

Video of F. nucleatum infection at 6 hours

Video of F. nucleatum infection at 24 hours

Video of F. nucleatum infection at 48 hours

Supplementary Table 1

Supplementary information

Figure S1

Figure S2

Figure S3

Figure S4

Figure S5

Supplementary Table 2
