# Supplementary material for: Host-microbe computational proteomic landscape in oral cancer revealed key functional and metabolic pathways between Fusobacterium nucleatum and cancer progression
Source: Int J Oral Sci. 2025 Jan 2;17:1. doi: 10.1038/s41368-024-00326-8 (PMC11693762; doi:10.1038/s41368-024-00326-8)
Supplement: Supplementary file 4 — Supplementary Table 1 [file 41368_2024_326_MOESM4_ESM.docx]

Table 1

| **Table 1 Patient Data** | | | | |
| --- | --- | --- | --- | --- |
| **Control** | | | | |
| **Age** | **Gender** | **Diagnosis** | **Patient ID** |  |
| 71  55  65  54  61  43  33  42  67  50  30  25  25  72  58  72 | Female  Male  Female  Female  Female  Male  Male  Male  Female  Female  Female  Male  Female  Female  Male  Male | Conjunctival epithelial hyperplasia  Conjunctival epithelial hyperplasia  Conjunctival epithelial hyperplasia  Conjunctival epithelial hyperplasia  Conjunctival epithelial hyperplasia  Healthy gum  Healthy gum  Fibrous hyperplasia  Conjunctival epithelial hyperplasia  Conjunctival epithelial hyperplasia  Fibrous hyperplasia  Healthy gum  Healthy gum  Conjunctival epithelial hyperplasia  Conjunctival epithelial hyperplasia  Conjunctival epithelial hyperplasia | CO-06  CO-07  CO-08  CO-09  CO-010  CO-013  CO-015  CO-016  CO-019  CO-020  CO-023  CO-026  CO-027  IHC-04  IHC-04  IHC-04 |  |
|  |  |  |  |  |
|  |  |  |  |  |
|  |  |  |  |  |
|  |  |  |  |  |
|  |  |  |  |  |
|  |  |  |  |  |
|  |  |  |  |  |
|  |  |  |  |  |
|  |  |  |  |  |
|  |  |  |  |  |
|  |  |  |  |  |
|  |  |  |  |  |
|  |  |  |  |  |
|  |  |  |  |  |
|  |  |  |  |  |
| **OSCC** | | | | |
| **Age** | **Gender** | **Diagnosis** | **Stage** | **Patient ID** |
| 73  88  76  56  70  66  73  74  66  58  76  67  76  70  74 | Male  Male  Male  Male  Male  Male  Male  Male  Male  Male  Female  Male  Male  Male  Male | Well-differentiated squamous cell carcinoma  Well-differentiated squamous cell carcinoma  Moderately differentiated squamous cell carcinoma  Well-differentiated squamous cell carcinoma  Moderately differentiated squamous cell carcinoma  Moderately differentiated squamous cell carcinoma  Moderately differentiated squamous cell carcinoma  Moderately differentiated squamous cell carcinoma  Moderately differentiated squamous cell carcinoma  Moderately differentiated squamous cell carcinoma  Well-differentiated squamous cell carcinoma  Well-differentiated squamous cell carcinoma  Moderately differentiated squamous cell carcinoma  Well-differentiated squamous cell carcinoma  Moderately differentiated squamous cell carcinoma | T2N1M0/II B  T2NOM0/II A  T4N2M0/III A  T3N1M0/III A  T2N2M0/III A  T3 N2b MX/III A  Unknown  Unknown  T1N0M0  T4aN0M0/IIIB  T1N0M0/I  T2N0M0/II A  T3N2M0/III C  T1N0M0/I  Unknown | CO-06  CO-07  CO-08  CO-09  CO-010  CO-013  CO-015  CO-016  CO-019  CO-020  CO-026  CO-027  IHC-04  IHC-04  IHC-04 |
