# Supplementary material for: Host-microbe computational proteomic landscape in oral cancer revealed key functional and metabolic pathways between Fusobacterium nucleatum and cancer progression
Source: Int J Oral Sci. 2025 Jan 2;17:1. doi: 10.1038/s41368-024-00326-8 (PMC11693762; doi:10.1038/s41368-024-00326-8)
Supplement: Supplementary file 5 — Supplementary information [file 41368_2024_326_MOESM5_ESM.docx]

**Supplementary**

**Supplementary Figure 1. A)** Venn diagram of bacterial peptides from malignant and healthy samples, showing the common and unique proteins for each condition. **B)** Heatmap of the profile of differentially expressed peptides from healthy and malignant samples.

**Supplementary Figure 2. A)** Analysis of cell death in HSC-3 cells the presence of Imidazole Ketone Erastin (IKE). **B)** Analysis of cell death in in HSC-3 cells in the presence or absence of Cystine. **C)** Analysis of cell death in in HSC-3 cells in the presence or absence of *F. nucleatum* with or without Imidazole Ketone Erastin (IKE).

**Supplementary Figure 3.** 3D Images of *F. nucleatum* inside HSC-3 cells, at 6 (**A**), 24 (**B**) and 48 (**C**) hours post infection.

**Supplementary Figure 4**. Representative photographs (10x) of tumorspheres from uninfected or *F. nucleatum*-infected HSC3 cells at day 3, 6 and 10.

**Supplementary Figure 5.** (**A**) Illustrative representation of the lysate and supernatant blots of uninfected or *F.* *nucleatum*-infected HSC3 cells in monolayer (24 hours). Bar charts of normalized protein expression of E-cadherin, ICAM-1(**B**), MMP3 and MMP9(**C**).
