# Supplementary figures and images for: Host-microbe computational proteomic landscape in oral cancer revealed key functional and metabolic pathways between Fusobacterium nucleatum and cancer progression
Source: Int J Oral Sci. 2025 Jan 2;17:1. doi: 10.1038/s41368-024-00326-8 (PMC11693762; doi:10.1038/s41368-024-00326-8)

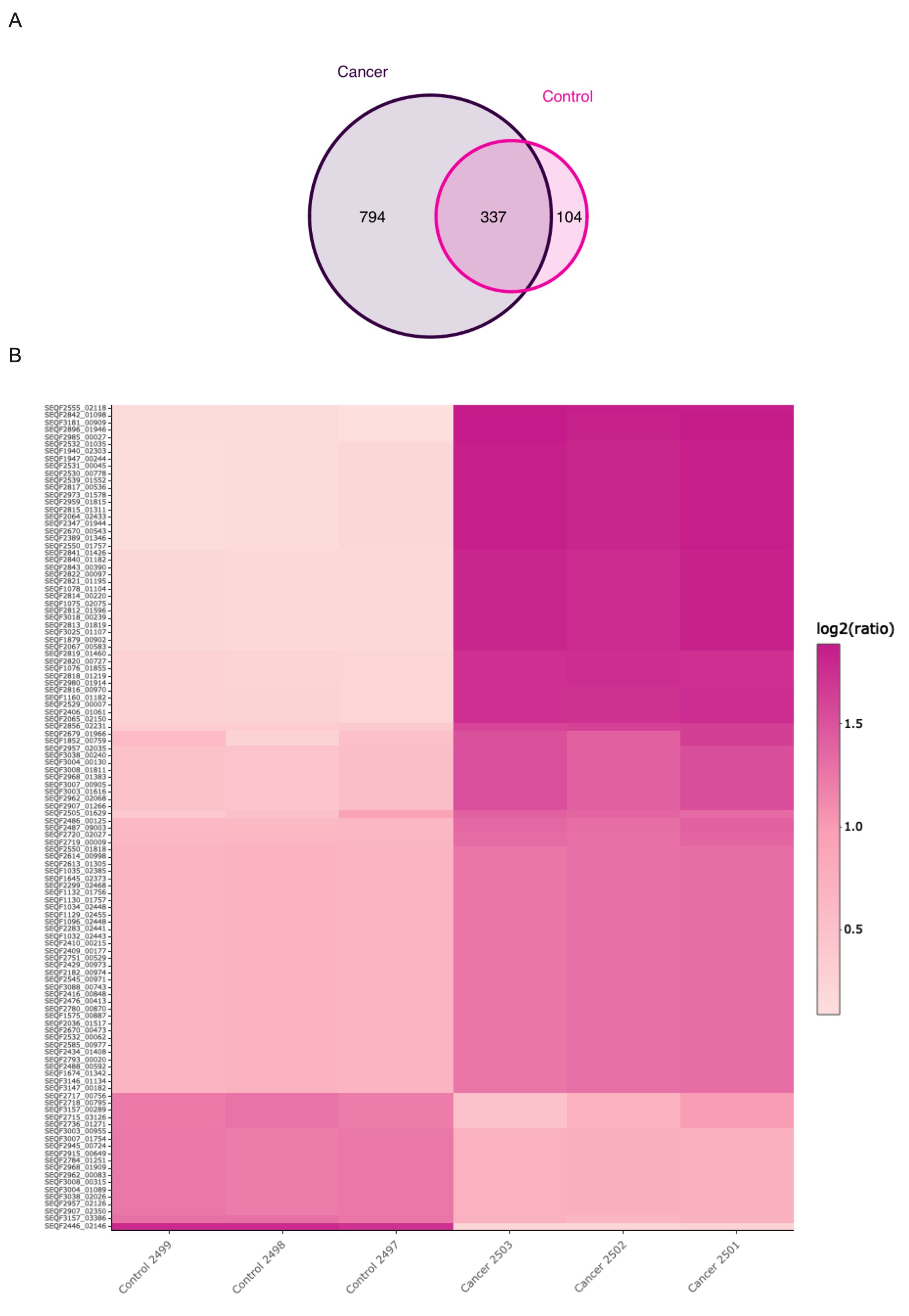

Supplement: Supplementary file 6 — Figure S1 [file 41368_2024_326_MOESM6_ESM.png]

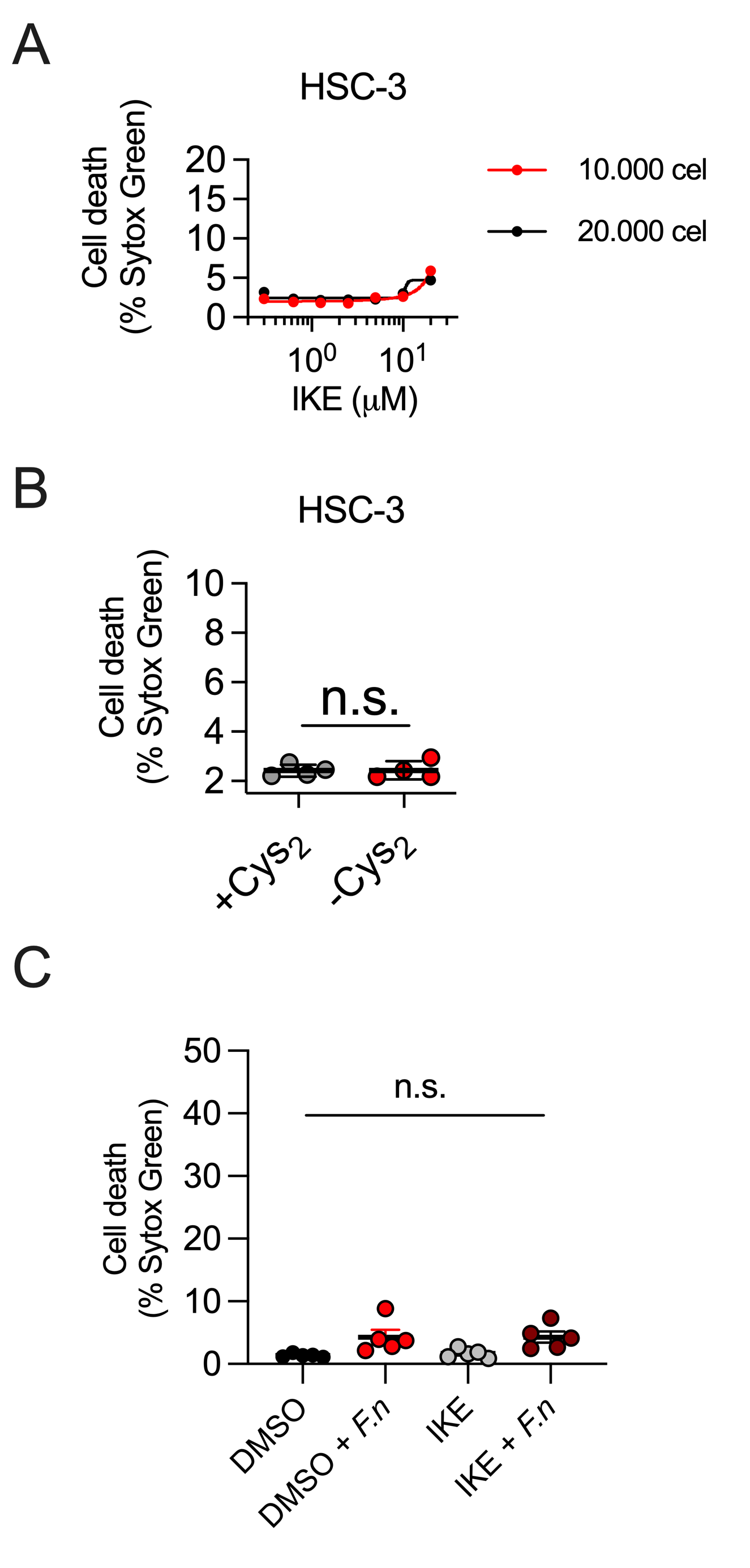

Supplement: Supplementary file 7 — Figure S2 [file 41368_2024_326_MOESM7_ESM.png]

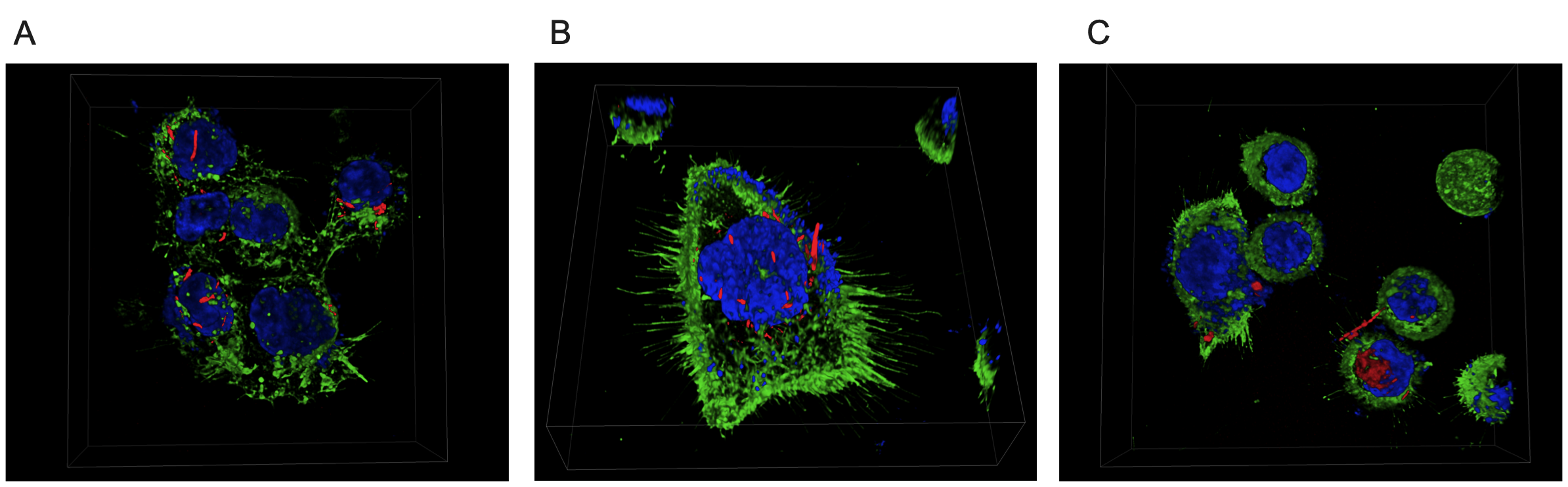

Supplement: Supplementary file 8 — Figure S3 [file 41368_2024_326_MOESM8_ESM.png]

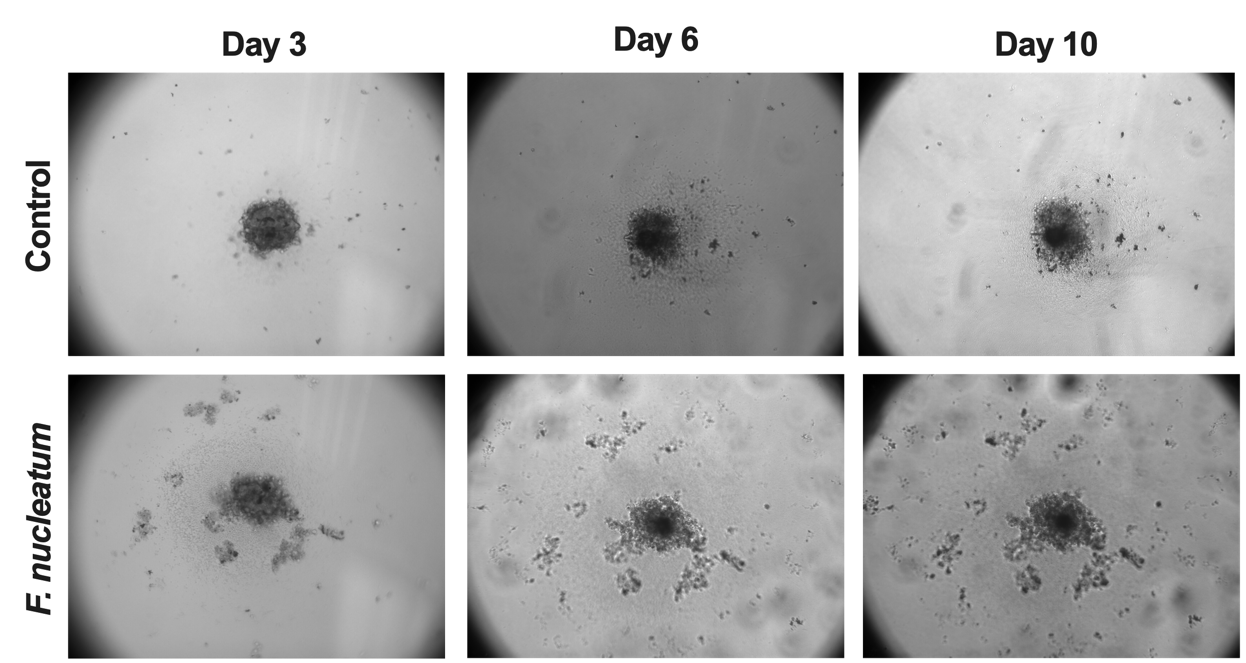

Supplement: Supplementary file 9 — Figure S4 [file 41368_2024_326_MOESM9_ESM.png]

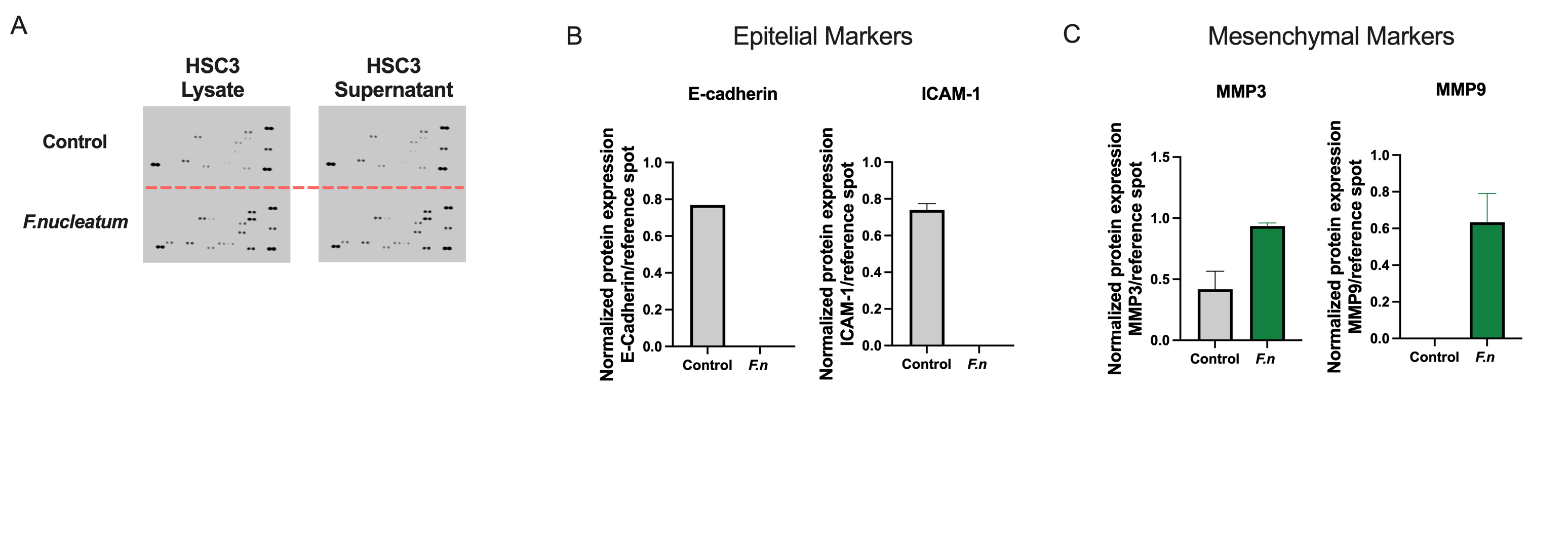

Supplement: Supplementary file 10 — Figure S5 [file 41368_2024_326_MOESM10_ESM.tif]
